# Metabolic syndrome in reproductive age: A cross-sectional study

**DOI:** 10.18502/ijrm.v23i5.19262

**Published:** 2025-07-29

**Authors:** Raisa Aringazina, Nurgul Zholdassova, Gulshara Berdesheva, Zhansulu Nurgaliyeva, Gulnara Kurmanalina, Bakhyt Zhanalina, Zoia Sharlovych

**Affiliations:** ^1^Department of Internal Diseases no. 1, West Kazakhstan Marat Ospanov Medical University, Aktobe, the Republic of Kazakhstan.; ^2^Department of Therapeutic and Orthopedic Dentistry, Khoja Akhmet Yassawi International University, Turkestan, the Republic of Kazakhstan.; ^3^Department of General Hygiene, West Kazakhstan Marat Ospanov Medical University, Aktobe, the Republic of Kazakhstan.; ^4^Department of Pharmacology, Kazakhstan-Russian Medical University, Almaty, the Republic of Kazakhstan.; ^5^Department of Internal Diseases no. 2, West Kazakhstan Marat Ospanov Medical University, Aktobe, the Republic of Kazakhstan.; ^6^Department of Surgical and Pediatric Dentistry, West Kazakhstan Marat Ospanov Medical University, Aktobe, the Republic of Kazakhstan.; ^7^Department of Pedagogy, International University of Applied Sciences in Lomza, Lomza, Poland.; ^8^Department of Professional and Pedagogical, Special Education, Andragogy and Management, Educational and Research Institute of Pedagogics, Zhytomyr Ivan Franko State University, Zhytomyr, Ukraine.

**Keywords:** Metabolic syndrome, Abdominal obesity, Dyslipidemia, Insulin resistance, Sex hormones.

## Abstract

**Background:**

Obesity and metabolic syndrome (MetS), as components of excess body weight, have reached global epidemic levels.

**Objective:**

The study aimed to investigate the features of the MetS course in men and women of reproductive age.

**Materials and Methods:**

This cross-sectional study was conducted with 140 participants aged between 35 and 49 yr at City Polyclinics No. 1, 3, and 4, Aktobe, Kazakhstan from March 2016–2019. Participants were divided into 2 subgroups based on diagnosis of MetS. The case group (with MetS), and the control group (without MetS) (n = 70/each consisted of 40 women and 30 men). Anthropometric, instrumental, and laboratory methods were used to confirm the diagnosis of MetS and determine its features in both groups.

**Results:**

The results showed an increase in the uric acid level in the blood to 431.00 ± 66.37 μ mol/L and 5.47 ± 0.90 mol/day in the urine. Dyslipidemia was also noted in the case group of individuals with high-density lipoprotein levels of 1.22 ± 0.27 mmol/L vs. 1.32 ± 0.47 mmol/L in the control group. The testosterone level was 16.4 ± 3.04 nmol/L in the case group and 20.3 ± 5.6 nmol/L in the control. The difference in testosterone level was found to be statistically significant (p < 0.01).

**Conclusion:**

The analysis of sex hormone levels did not reveal any stable trends that could be considered diagnostic. That may indicate a correlation with the reproductive age characteristics of the examined individuals (men and women): significant differences between hormone levels were fixed in the case and control groups.

## 1. Introduction

Infertility affects about 15% of couples worldwide, and only 40–50% of them can be successfully treated (1). There is evidence indicating that individuals with obesity face more significant obstacles to conception. Fortunately, overweight is correctable, and overweight infertility can be treated (2, 3). According to a World Health Organization (WHO) report, over a billion people worldwide are susceptible to the disease (body mass index [BMI] ≥ 30 kg/m^2^), and 650 million of them are reproductive-age adults. Approximately 1.9 billion people are classified as overweight (4, 5). Obese people need medical care even after weight loss, as excessive fat accumulation can cause metabolic disorders and activate inflammatory mechanisms (3, 6).

The progression of obesity is usually associated with metabolic syndrome (MetS) (7). Beigh and Jain studied the features of the MetS development in the citizens of the United States. They noted the prevalence of MetS development in women vs. men (8). Their data were in accordance with previous research in Russian, Korean, and Chinese populations. Moreover, Nilsson et al. (9) have proved that the effect of the genetic predisposition and hormonal imbalances in MetS development is especially relevant for women.

It has been fixed that MetS has some common and different features in men and women, which are related to physiological features in genders (8–10). Hyperglycemia and insulin resistance are typical features in MetS progression and development of diabetes mellitus (DM). Hyperglycemia is related to the cellular (cellular senescence) and molecular dysfunction development because of the increasing oxidative stress (OS) which contributes to the production of advanced glycosylation end products (11). Also, common symptoms for MetS can be named atherogenic lipid disorders, hyperuricemia, abdominal obesity (AO), and arterial hypertension (9, 10).

When diagnosing MetS, it is necessary to consider a combination of symptomatic disorders. The issue of MetS is not confined to a specific area. Therefore, authoritative health organizations such as the American Heart Association (12), the National Cholesterol Education Program (13), and the Diabetes Federation considered this problem and made recommendations. In this way, clinicians have to take care of the following symptoms as AO (if the waist circumference [WC] values of > 40 inches in men and > 35 inches in women); the level of triglyceride (TG) (150 mg/dL) or consumption of TG-lowering drugs; high-density lipoprotein (HDL) level < 40 mg/dL (for man) and < 50 mg/dL (for women); elevated low-density lipoproteins > 3.0 mmol/l; hypertension (blood pressure [BP] > 130/85 mm Hg), and hyperglycemia (fasting blood glucose level ≥ 100 mg/dL), impaired glucose tolerance (2 hr post-load plasma glucose levels of ≥ 7.8 mmol/l but < 11.1 mmol/l); waist-to-hip ratio (WHR) of ≥ 0.85 for women and ≥ 0.9 for men; (12–14). 2 or more (13, 14) or 3 or more (12) signs must be detected in a patient to make a MetS diagnosis.

Silveira Rossi et al. (11) noted the impact of obesity accompanied by the MetS on cardiovascular disease (CVD) development. Of course, these diseases should not be considered as totally triggered by MetS, but metabolic disorders make a higher CVD risk development due to the progression of atherosclerosis. Moreover, CVD's progression is associated with OS and inflammation in the body that rises due to overaccumulation of adipose tissue (11, 15). Obesity and high cholesterol levels contribute to the accumulation of cholesterol under the vascular epithelium layer, which causes vascular narrowing or occlusion. This process activates the inflammatory development that makes the vascular lumen smaller, too. Later, the inner walls of the epithelium of the coronary arteries can be injured. This leads to an increase in pressure against the coronary artery and worsens the lifestyle of the overweight person (15).

Naturally, the biggest differences are related to the sex hormone levels in men and women with MetS. By analyzing women's fertility, we can note the reduction in women's chances of successful impregnation (2) due to infertility, polycystic ovarian syndrome, low estrogen levels and elevated testosterone, endometriosis development and OS progressing (16, 17). Men with MetS have a risk of experiencing decreased libido and reduced chances of insemination (2) due to hypogonadism, diminished libido, erectile and ejaculatory dysfunction, reduced sperm motility, low levels of testosterone, and follicle-stimulating hormone (18, 19).

The study aimed to identify the characteristics of MetS factors in reproductive-age, observed in males and females by testing sex hormone levels in individuals with MetS and assessing the influence of MetS on reproductive function in the fertile-age population.

## 2. Materials and Methods

### Participants and study design

This cross-sectional study was conducted on 140 participants aged between 35 and 49 yr at City Polyclinics No. 1, 3, and 4, Aktobe, Kazakhstan from March 2016-March 2019. Participants were divided into 2 subgroups based on diagnosis of MetS.

The case group (n = 70) consisted of 40 women and 30 men with MetS, and the control group consisted of 40 women and 30 men without MetS.

According to the WHO, the reproductive age is between 15 and 49 yr (20) that we took as a base. However, due to national features, the overweight of the Kazakh population becomes apparent for inhabitants over 30, with the top age being 42–49 (21). Hence, we majorly focused on the participants over 30 yr, and most participants included in our research were from 35–49.

Anthropometric (body sizes and indexes), instrumental (BP), and laboratory (biochemical and hormonal hematology research) methods were used to confirm the diagnosis of MetS and determine its characteristics (clinical variations and potential pathogens) and mechanisms in participants.

The parameters of lipid, carbohydrate, and purine metabolism were evaluated to determine their values' depending on BMI and WHR. The sex-specific hormone levels, estradiol in women and testosterone in men, have been analyzed to identify possible patterns and diagnostic dependencies.

The inclusion criteria were as follows: 1) age from 35–49 yr (both men and women in reproductive age); 2) the absence of critical, chronic, or incurable diseases such as oncology of all stages, organ and multiple failures, mental disorders, or decompensated endocrine pathologies.

The exclusion criteria were as follows: 1) pregnancy; 2) treatment by glucocorticosteroids; 3) presence of an acute contagious disease.

### MetS diagnostic criteria

MetS was diagnosed based on the following criteria: WC > 80 cm for women and > 94 cm in men; WHR > 0.85 for women and > 0.9 in men; BMI > 30 kg/m^2^; BP > 130/85 mmHg in systolic/diastolic phases; and TG rate > 1.7 mmol/L (12–14). A MetS diagnosis is made if a person has been diagnosed with a combination of 3 (or more) criteria.

Anthropometric measurements and instrumental and laboratory tests were performed under Public Procurement Services Contract No. 211, dated 2016–04-06.

### Anthropometric testing

All participants were measured for height, body weight, waist and hip circumferences with the help of a mechanical measuring tape RP `Econom' (JSC Tulinovsky Instrument-Making Plant TVES, Russia) and scales VEM-150-`Massa-K' (MASSA-K, Russia). A tape with a double scale (200 cm) was used to measure the waist and hips.

Obesity was determined based on the BMI, calculated as follows: $\text{BMI}=\frac{\text{m}}{h^{2}}$ where m is the body weight in kilograms; *h* is the height in meters. Adipose tissue distribution was determined based on WHR values. 


$$\text{WHR}=\frac{\text{WC}}{\text{WH}}$$

If the WHR is ≥ 0.85 in women or ≥ 0.9 in men, the AO was considered (according to NCEP-ATP III) (15). However, it has to be considered that according to the International Diabetes Federation (IDF) (16), AO is diagnosed in women with WC > 80 cm and in men with WC > 94 cm.

### Laboratory and instrumental testing

5 ml of blood sample from each patient was collected and tested in the laboratory `Invitro' (Aktobe, Republic of Kazakhstan). Blood plasma was separated from the blood sample after the clot was completely formed, then it was centrifuged at 3000 rpm for 10 min. Serum was stored at -80 C until the time of analysis. Biochemical parameters were tested by the Architect c8000 Clinical Chemistry Analyzer (Abbott, USA).

#### BP measurements

Systolic and diastolic BP were measured by using the tonometer-aneroid `Microlife BP AG1–30' (Microlife Corp., Switzerland) at each examination using Korotkoff sounds (right hand) after a 5-min rest, at least 3 times, with the average value calculated. NCEP-ATP III and IDF guidelines were used to diagnose hypertension in combination with MetS (13, 14). Thus, hypertension was diagnosed with systolic and diastolic BP values of > 130/85 mm Hg.

#### Carbohydrate metabolism status

Carbohydrate metabolism was assessed utilizing the Carbohydrate Metabolism Test kit. Blood glucose levels were measured using an enzymatic colorimetric assay (glucose oxidase method [GOD-PAP]). Blood glucose levels were measured using an enzymatic colorimetric assay GOD-PAP. Venous blood samples (5 mL) were taken in the morning after fasting, and the normal glucose level in blood serum was 3.5–5.6 mmol/L.

Oral glucose tolerance and C-peptide tests were performed at glucose levels above 5.6 mmol/L. Our tests were based on the IDF criteria (14). Normoglycemia was defined as glucose levels < 6.1 mmol/L and > 7.8 mmol/L after 2 hr. Hyperglycemia was defined as glucose levels 6.1–7.0 mmol/L and > 7.8 mmol/L after 2 hr. Furthermore, impaired glucose tolerance was diagnosed when the glucose level was ≤ 7.0 mmol/L and 7.8–11.1 mmol/L in 2 hr.

#### Insulin level and sensitivity tests

It is worth noting that insulin concentrations in the bloodstream can vary significantly in healthy individuals. The variability can be attributed to individual differences in nervous system type, ethnicity, and lifestyle. This fact underscores the need for caution when adjusting insulin levels. We used the upper quartile as the threshold reference value for insulin resistance, and insulin sensitivity was measured in the lower quartile of the indicators (22). Insulin levels were determined in serum (by immunoassay), strictly on an empty stomach (more than 8 hr between the last meal and the blood drawn).

For DM risk assessment, the glycated hemoglobin (HbA1c) level was measured by chemiluminescent immunoassay, which is based on the American Diabetes Association guidance (23).

#### Lipid spectrum of blood serum

TG and HDL cholesterol levels were measured by colorimetric assay (homogeneous enzymatic method) in blood collected after a 12 hr fast. Dyslipidemia was assessed according to the WHO criteria (24). The following values were obtained: TG concentrations > 1.7 mmol/L and HDL < 1.0 mmol/L for women and < 0.9 mmol/L for men.

#### Protein metabolism's parameters checking

The uric acid in the blood and urine levels were measured, given the reference parameters for serum of 150–350 μ mol/L in women and 210–420 μ mol/L in men. For urine, the reference value was 1.48–4.43 mmol/L.

#### Sex hormonal state detection

The sex hormones (estradiol and testosterone) levels were determined by enzyme-linked immunosorbent assay to analyze the fertile state under the American Association of Clinical Endocrinologists guidelines (25).

### Sample size

The sample size was calculated based on the average annual number of referrals to the medicals with MetS complaints to determine statistical significance. The Fleiss method was chosen for our research in forming the study groups.


$$N_{Fleiss}=\frac{[z_{a/2}\sqrt{\left(r+1\right)p\left(1-p\right)+Z_{\beta }\sqrt{rp_{0}\left(1-p_{0}\right)+p_{1}\left(1-p_{1}\right)}{]}^{2}}}{r{\left(p_{0}-p_{1}\right)}^{2}}$$

Where $Z_{a/2}$ is the standard normal deviate for a 2-tailed test based on alpha level (relates to the confidence interval level); $Z_{\beta }$ -standard normal deviate for one-tailed test based on beta level (relates to the power level); r is the ratio of unexposed to exposed; $p_{1}$ is a proportion of case group; and $p_{0}$ -the proportion of the control group.

A total of 140 participants were included in the research with the ratio of controls to cases of 1:1 (70 case patients and 70 control members) and a 2-sided confidence level of 95%. The percentage of controls exposed was set at 20% and power at 80%. However, we crossed the point that the probability of detecting decrease in fertility can be detected in 80% of patients with diagnosed MetS (if this relation is present) (26).

### Ethical Considerations

The study was ethically approved by the Bioethics Committee of West Kazakhstan Marat Ospanov State Medical University, Aktobe, the Republic of Kazakhstan, which confirmed the experiment's compliance with the Declaration of Helsinki (1964) (Code: 8/4–1-178502). All participants provided written informed consent to participate in the de-identified study and to have personal data processed.

### Statistical Analysis

The clinical laboratory test results were analyzed using Statistical Package for data analysis, management, mining, and visualization by statistical methods STATISTICA, version 10.0 (TIBCO Software Inc., USA). The differences were considered statistically significant at p ≤ 0.05. Median mean (Me) and interquartile range (IQR), which describe the measure of statistical dispersion, were measured. Moreover, median/upper quartile/lower quartile was additionally calculated for variables with non-normal distribution. The Shapiro-Wilk test was used to check the normality of the data distribution. According to checking data normality, Mann-Whitney U-test and Student's *t* test were used for analysing the data of the case and control groups: Mann-Whitney U-test was used for non-normality data distributed and Student's *t* test for normality distributed data. To analyze the relation between variables and check the interrelation between the variables has been conducted by using Spearman's rank correlation coefficient (27).

## 3. Results

### Characteristics of the involved participants: Principle of forming examined groups

The case and control groups were formed based on the results of anthropometric measurements, instrumental and laboratory tests, and the recommendations for diagnosing MetS (21, 24, 25). The case group (participants with MetS) was equivalent to the control and involved 70 people (40 women and 30 men) of same age group. First of all, we had the goal to prove the presence of the MetS in patients of the case group and the absence of such signs combination in the members of the control group (equivalent in participants number and age group), as outlined in table I.

**Figure 1:**
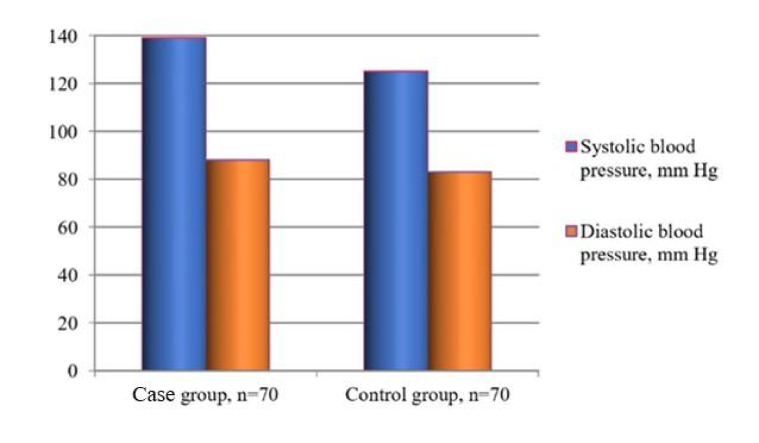
BP levels in the study participants.

Thus, the initial tests revealed that the case group participants had AO (BMI > 30 kg/m^2^) and hypertriglyceridemia. Many of them also had hypertension 139 ± 17/88 ± 1.3 mm Hg (Qmin = 130/85; Qmax = 142/90) (Figure 1), which allowed for the diagnosis of MetS. Noteworthy, the combination of these parameters was not detected in the control group patients 125 ± 0.30/83 ± 0.74 mm Hg (Qmin = 120/80; Qmax = 130/85 mm Hg).

So, participants of the case group demonstrated a general tendency toward hypertension, which was not observed in the control group.

### Anthropometric measurements

**Figure 2:**
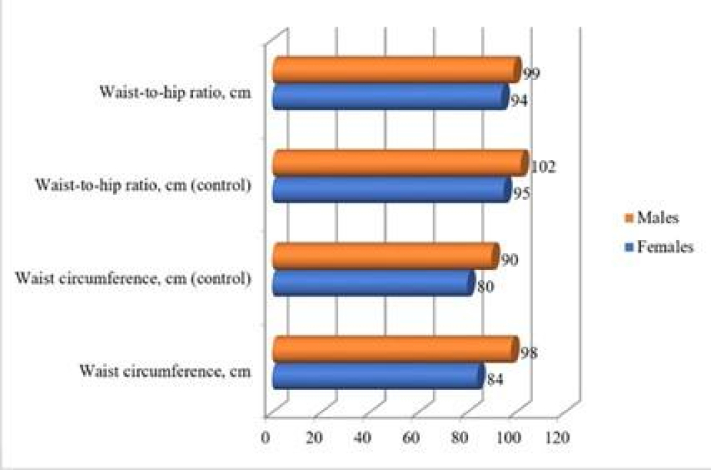
Correlation of anthropometric measurements.

BMI testing exhibited that the average data in both groups exceeded 30 kg/m^2^ corresponding to obesity development. Furthermore, the case group's BMI was higher: 32.5 ± 1.5 kg/m^2^ compared to the 30.5 ± 0.7 kg/m^2^ in the control one. According to the Shapiro-Wilk test, the most of the anthropometric data were normally distributed, which excluded hip and waist measures of the man's case group. It should be noted that despite class 1 obesity in several control group participants, they were not diagnosed with MetS. Figure 2 illustrates the correlation of anthropometric parameters of the control and case groups according to gender distribution.

The body fat distribution analysis indicates that women in both groups manifested signs of AO development: WHR of 0.88 in the case group against 0.85 in control group, which can be interpreted as the AO presence (threshold value: 0.85). The threshold value (0.9) indicated that AO development among the men was also observed in the control group. WHR in the case group significantly exceeded the 0.96 threshold value. Nonetheless, it is significant that the AO presence is an indicative but insufficient symptom for diagnosing MetS. For example, several control participants with obesity did not have the associated symptoms (hypertension, dyslipidemia) required for MetS diagnosis (at least 3 symptoms in total).

### Assessment of the metabolic features in the control and case groups 

As part of the research, we assessed carbohydrate metabolism in individuals with MetS and control participants who were not diagnosed with MetS despite having partial class 1 obesity. Checking the normality distribution according to the Shapiro-Wilk test fixed p < 0.05, which indicated non-normality data distribution. So, non-parametric test was used for statistical analysis of the non-normality distributed data. A significant difference was observed between analyzed parameters in the groups according to non-parametric Mann-Whitney U-test (p < 0.05) (Table I).

The carbohydrate metabolism analyzed, suggests a risk of diabetes development in analyzed patients. According to the enzymatic colorimetric assay, elevated glucose levels were noted for the case group. However, it was not a total tendency (5.80 ± 0.58 mmol/L). The same state was fixed for the insulin levels (Table I). The average HbA1c levels (a stable compound formed by the binding of glucose molecules to hemoglobin) were also higher in the case group. And for some patients, DM becomes a real damage: 5.50 ± 0.88% for case group vs. 5.00 ± 0.56% for control.

### Lipid metabolism indicators

A negative ratio (r = -0.5; p < 0.05) was found between the decrease in HDL mmol/L (1.09 ± 0.42 mmol/L) and the increase in TG average levels (2.09 ± 0.40 mmol/L) for the case group (Table II). Also, it should be noted that the TG content was significantly higher (p < 0.05) in the case group compared to the control.

### Indicators of protein metabolism

The uric acid concentration analysis in blood, an indicator of protein metabolism, revealed that its level in the case group increased to 431.00 ± 66.37 μ mol/L (at a normal level in blood serum 178–345 μ mol/L); while in urine, it was 5.47 ± 0.90 mol/day with reference levels of 1.48–4.43 mol/day. For the participants of the control group, these indexes were in the reference levels: 286 ± 53.7 μ mol/L and 3.8 ± 0.47 mol/day.

### Assessment of the hormonal profiles

**Figure 3:**
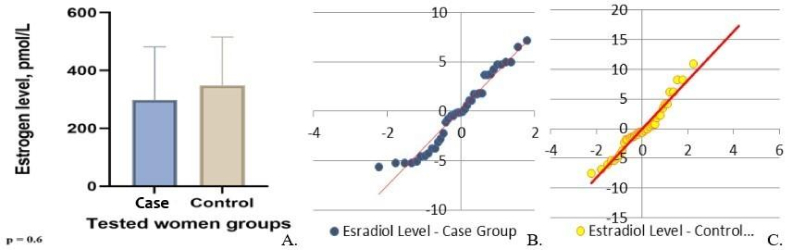
Estradiol levels in the female participants of the case and control groups: A) Medium concentration, B) Distribution of the gotten variables in the main (case) group, C) Distribution of the gotten variables in the control group.

Shapiro-Wilk test demonstrated non-normality distribution for the variables of the estradiol levels for both groups. The estradiol level in females was checked, considering the difference in estradiol reference values depending on the woman's cycle phases: follicular phase-68-1269 pmol/L; ovulatory phase-131-1655 pmol/L; luteal phase-91-861 pmol/L. The average estradiol level in the women from the case group was 291.8 ± 307 pmol/L. The minimum serum estradiol concentration was observed in a 49-yr-old woman, with a maximum peak reported in a 48-yr-old. The minimum and maximum estradiol concentrations in the control group were 31 pmol/L (48 yr) and 783 pmol/L (45 yr), respectively. The average value was equal to 327 ± 146.50 pmol/L (Figure 3).

The findings revealed that indicators for both groups differed insignificantly and were within a reference range for the corresponding phases of the cycle. Therefore, we may assume that a decrease in estradiol concentration depends on the individual characteristics of the female hormonal system and requires a more profound study. While testing Student's *t* test, we noted the absence of significant difference; however, while using Mann-Whitney U test for non-parametric test, we discovered the difference between tested person's groups.

In checking the estradiol levels between groups under the Mann-Whitney U test, we had a fixed U-statistic of 8284.0 and a p = 2.99 × 10⁻⁵ which means highly significant difference between case and control groups.

**Figure 4:**
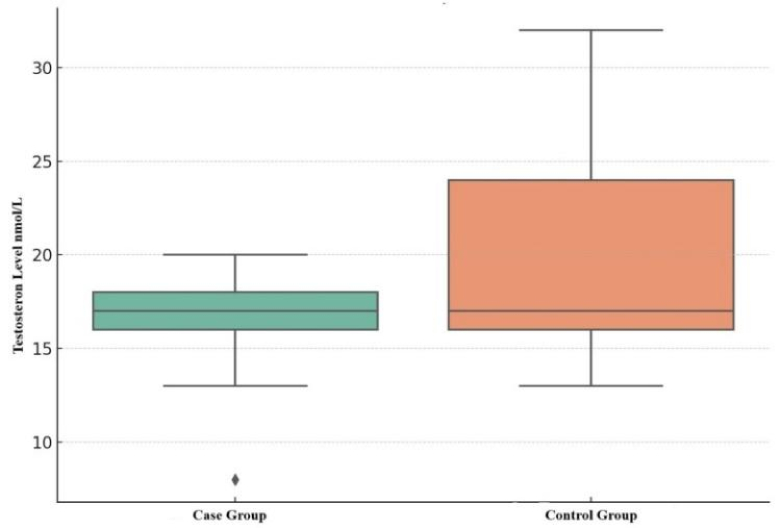
Blood testosterone levels in male participants of the case and control groups.

The hormonal profile analysis of the male samples showed that the variables had not only the line distribution but also normality distribution. The testosterone levels were in the reference values (8.9–42 nmol/L) in both groups: 17.00 ± 2.00 in the control group and 18.00 ± 8.00 nmol/L in the control participants. However, levels of the case group were significantly lower than in the control (p < 0.01) (Figure 4): Qmin = 8 nmol/L and Qmax = 20 nmol/L in the case; and Qmin = 13 nmol/L and Qmax = 32 nmol/L in control participants.

We used Mann-Whitney U-test to check the testosterone levels between case and control groups. According to the gotten data, U-statistic was near to 0 and p-value was fixed at the near level of 7.50 × 10⁻³¹ which signalled about an extremely significant difference between tested groups.

Therefore, we can note the low p-values in checking both estrogen and especially testosterone levels. That is why we indicated significant differences between the case and control groups.

It is essential to underscore the complexity of the topic by mentioning the various factors that need to be considered in further investigation into the fertility of men with MetS, including their psychological state, temperament, and hormone profile.

**Table 1:** Indicators of carbohydrate metabolism in individuals with MetS

**Indicators**	**Reference values**	**Case group (n = 70)**	**Control group (n = 70)**	**P-value**
Glucose (mmol/L)*	5.6	5.80 ± 0.58	5.20 ± 0.10	< 0.001
Insulin (μU/ml)*	2.6–24.9	24.95 ± 4.68	10.00 ± 1.00	< 0.001
HbA1c (%)**	≥ 6.5	5.50 ± 0.88	5.00 ± 0.56	< 0.001
The data is presented in the format of Median ± IQR. The normality of the data distribution was checked by the Shapiro-Wilk test, *Mann-Whitney U-test has been used for not normally distributed data. **Normality distribution has been fixed only for the case group. That is why Mann-Whitney U test was also used for this parameter analysis. MetS: Metabolic syndrome, HbA1c: Glycated hemoglobin				

**Table 2:** Comparative indicators of the blood lipid spectrum in the examined individuals

**Indicators**	**Reference values**	**Case group (n = 70)**	**Control group (n = 70)**	**P-value**
HDL (mmol/L)*	≥ 1.3	1.09 ± 0.42	1.29 ± 0.01	< 0.0001
TG (mmol/L)*	≤ 1.7	2.09 ± 0.40	3.40 ± 0.2	< 0.05
The data is presented in the format of Median ± IQR. The normality of the data distribution was checked by the Shapiro-Wilk test, *Mann-Whitney U test was used for not normally distributed data. HDL: High-density lipoproteins, TG: Triglycerides				

## 4. Discussion

The current cross-sectional research with 2 subgroups was aimed to study the impact of obesity and related MetS on adult fertile Kazakh individuals. First of all, we have proved the presence of the MetS in the patients of the case group. MetS was diagnosed in participants with a combination of 3 or more critical parameters presented in table I, and we have to note that a single obesity presence fact does not mean the MetS. We have studied metabolic, anthropometric, and physiological features by analyzing lipid and carbohydrate metabolism with checking diabetes-related indexes (insulin and HbA1c), and physiological parameters such as BP, which is important to the whole body's functionality. However, the main task of our research was to analyze sex-hormonal levels in patients with MetS to provide a comprehensive assessment of the fertility implications for individuals with MetS. Moreover, we were looking for the impact of the MetS on the reproductive (hormone) system, for which we have checked estrogen and testosterone levels accordingly to the participants gender.

Biochemical haematological analysis noted impaired lipid and protein metabolisms for the case group participants. Also, the individuals with MetS had hyperuricemia, which may be related to Kazakh cultural and nutritional traditions, such as extensive consumption of red meat (28). Consequently, this indicator must be analyzed more thoroughly, especially the effect of other factors (different from MetS) on purine metabolism. We have not found significant relations in women's hormonal levels; however, the male hormonal state was significantly lower in the MetS group (p < 0.01 according the student's *t* test and p ≈ 7.50 × 10⁻³¹ according to the Mann-Whitney U-test). So, we can note that the sensitivity of the male hormonal state is greater compared to that of women in persons with MetS. However, we have to declare the need for a deeper study of women's hormonal levels in relation to the female physiological cycle.

MetS is related to obesity, which can be marked as a multifactorial disease since its pathogenesis is associated with several biological, psychosocial, socioeconomic, and environmental factors (29). One factor accelerating the progression of obesity is MetS, which is regarded as a series of shifts in metabolic processes that cause and intensify pathological changes in critical body systems (7). Therefore, we can state that our research is relevant in the world format, considering the WHO obesity data as the world's pandemic problem (4) and whole-world research (2, 5, 7, 11). We have to fix the relationship between the MetS and hypertension development: the most significant part of the participants with diagnosed AO had hypertension (Figure 1). Moreover, the patients experienced recovery from carbohydrate, lipid, and protein metabolism disorders. DM was not fixed in MetS group patients; however, we noted a tendency toward insulin resistance in this group, which can be regarded as a risk factor.

This theory was tested by determining the level of HbA1c as a diagnostic criterion for DM (14), considering the fact that DM progresses if the HbA1c level is 0 ≥ 5.50%, while HbA1c above 6.5% indicates a risk of DM complications (23). It was revealed that the HbA1c level was almost the same in both (case and control) groups. Thus, DM development can be taken as a threat in MetS patients. Nevertheless, it should be noted that high insulin, glucose, and HbA1c levels in the case group are still alarming signs. Even experimentally provoked short-term (105 min) hyperinsulinemia (hyperinsulinemia-euglycemia) elevates the concentration of inflammatory markers and β-amyloid peptide in cerebrospinal fluid and serum (30). Chronic euglycemic hyperinsulinemia (72–96 hr) is characterized by insulin resistance and impaired non-oxidative glucose metabolism. Thus, the consequences of this condition depend on the pathology's duration and intensity (31).

As one of the essential human life components, we were interested in fertility and the sex hormone levels in obese individuals and the control groups. This is because of data on obesity's impact on libido, impregnation, fertility, and pregnancy (11, 16): hyperinsulinemia in women leads to hormonal imbalance, hyperandrogenism, and polycystic ovaries which the adipose tissue features can explain to contribute to endocrine dysregulation regardless of gender (31). Lower levels of sex hormones were observed in the case group. Some data fixed parameters lower than the reference norms: 18 pmol/L was the lowest rate of estradiol in females with obesity. Nonetheless, stable diagnostic criteria have not been found for female groups (p = 0.6). The difference between male groups was significant: the testosterone level in the male group with MetS was significantly lower than that in the control. This can be explained by such obese tissue features as its impact on rising body temperature, OS development (that is, characteristics of both genders), and changes in erectile and ejaculatory functions (which can significantly reduce sperm production and motility) (5, 16).

Thus, it is crucial to conduct thorough research on the effects of obesity on a person's subfertility. This research should consider government and healthy lifestyle policy, social empathy, and an evidence-based medical approach. MetS patients require an individualized approach focused on improving their life quality and overall health rather than weight loss. At the same time, the national and cultural peculiarities of the region, as well as data about the traumatic experience of predisposed overweight persons, should be considered (2, 3, 16, 32).

### Strengths and limitations

We have researched the primary group of individuals diagnosed with obesity and MetS conditions, which comprised individuals aged between 35 and 49. Nevertheless, we still have to analyze the rest of the reproductive-age people.

Our research has not addressed women's cycle flow. Therefore, this aspect must be studied deeply to better understand the impact of MetS disorders on a person's fertility. The comparison of the infertility cases (appeals in clinics) and the MetS cases in these persons must be studied in the future. The interrelation between the sex hormone concentration and the progression of MetS should be studied in more detail. Also, one more aspect needs to be more thoroughly analyzed. An increased uric acid level in the blood (431.00 ± 66.37 μ mol/L) and urine (5.47 ± 0.90 mol/day) can be partially attributed to the food culture in the Republic of Kazakhstan.

## 5. Conclusion

Obesity accompanied by the MetS is a combined pathology that impacts all body systems and influences in person's physiological processes flowing, which changes a person's life quality. Today, it is a big problem in fertility for the whole world. MetS can be one of the reasons for infertility development, so we have analyzed biochemical, physiological, and hormonal parameters in persons with diagnosed MetS.

According to our results, people with MetS had protein, lipid, and carbohydrate metabolism disorders. Regardless, we recorded the absence of DM signs in the tested case group, but gotten laboratory results of the insulin and HbA1c levels indicated the need to correct and normalize carbohydrate metabolism in the MetS group. The testing of the sex hormone levels has demonstrated a negative impact of the MetS on reproductive function in the fertile-age population.

The analysis of the sex hormone levels in MetS males and females has not revealed stable trends that could be used as diagnostic (ranges were fixed in reference rates). Nevertheless, the difference in the case and control men groups was significant, p < 0.05. Analysis of the women's sex hormone ranges (estrogen) has indicated a vital difference between case and control groups according to the Mann-Whitney U test. However, due to more significant differences, we can conclude that the male hormone system was more sensitive to the influence of the obese-introduced changes in the human body. Nonetheless, we cannot mark the diagnostic parameters for relations between MetS and sex hormone levels.

##  Data Availability

All raw research materials can be presented according to the reasonable request of the corresponding author.

##  Author Contributions

R. Aringazina: Was a developer of the research design and conception, curated analyzing of the data processing, had a part in the manuscript creation, analysis, and paper correcting. N. Zholdassova: Had a part in research design creation, research data collecting and processing, and she was the creator of the methodology and results parts of the manuscript. G. Berdesheva: Had a part in the research data analysis and interpretation, created the discussion section of the manuscript. Zh. Nurgaliyeva: Had a part in the research data processing and was the main in statistical processing of the collected data; she was responsible for translating the raw manuscript text into English. G. Kurmanalina: Collected, analyzed, and had a part in interpreting research data, was responsible for the introduction section creation. B. Zhanalina: Had made previous review and critical analysis of the main part of the research, had a part in paper correction and creation. Z. Sharlovych: Analyzed and processed research data, made a previous review of the manuscript before sending it to the journal's edition. All of the authors read and approved the final variant of the manuscript before it was sent for consideration.

##  Conflict of Interest

The authors declare that there is no conflict of interest.
